# Impact of Early Life Stress on Reward Circuit Function and Regulation

**DOI:** 10.3389/fpsyt.2021.744690

**Published:** 2021-10-20

**Authors:** Jamie L. Hanson, Alexia V. Williams, Debra A. Bangasser, Catherine J. Peña

**Affiliations:** ^1^Department of Psychology, University of Pittsburgh, Pittsburgh, PA, United States; ^2^Department of Psychology and Neuroscience Program, Temple University, Philadelphia, PA, United States; ^3^Princeton Neuroscience Institute, Princeton University, Princeton, NJ, United States

**Keywords:** early life stress (ELS), reward, nucleus accumbens (NAc), ventral tegmental area (VTA), development, ventral striatum

## Abstract

Early life stress – including experience of child maltreatment, neglect, separation from or loss of a parent, and other forms of adversity – increases lifetime risk of mood, anxiety, and substance use disorders. A major component of this risk may be early life stress-induced alterations in motivation and reward processing, mediated by changes in the nucleus accumbens (NAc) and ventral tegmental area (VTA). Here, we review evidence of the impact of early life stress on reward circuit structure and function from human and animal models, with a focus on the NAc. We then connect these results to emerging theoretical models about the indirect and direct impacts of early life stress on reward circuit development. Through this review and synthesis, we aim to highlight open research questions and suggest avenues of future study in service of basic science, as well as applied insights. Understanding how early life stress alters reward circuit development, function, and motivated behaviors is a critical first step toward developing the ability to predict, prevent, and treat stress-related psychopathology spanning mood, anxiety, and substance use disorders.

## Introduction

Early life stress (ELS) increases lifetime risk of depression, suicide, and mood, anxiety, and substance use disorders, and epidemiological studies suggest that approximately 30% of all adult-onset psychiatric disorders are associated with the experience of ELS (1–8). Broadly, ELS includes a range of adverse to traumatic experiences, ranging from socioeconomic disadvantage to loss of a parent, institutionalization, neglect, abuse, or exposure to domestic or community violence. These stressful experiences can be categorized as related to deprivation (an absence of expected age-typical stimuli and experiences) or threat (presence or perceived risk of physical violation or harm) (9, 10). Timing and chronicity of ELS exposure may also impact psychiatric outcomes (11–14). Understanding how different forms, timing, or cumulative ELS exposure alter brain development and function to impart risk is essential in order to develop better interventions for this vulnerable population (15, 16).

**Figure 1:**
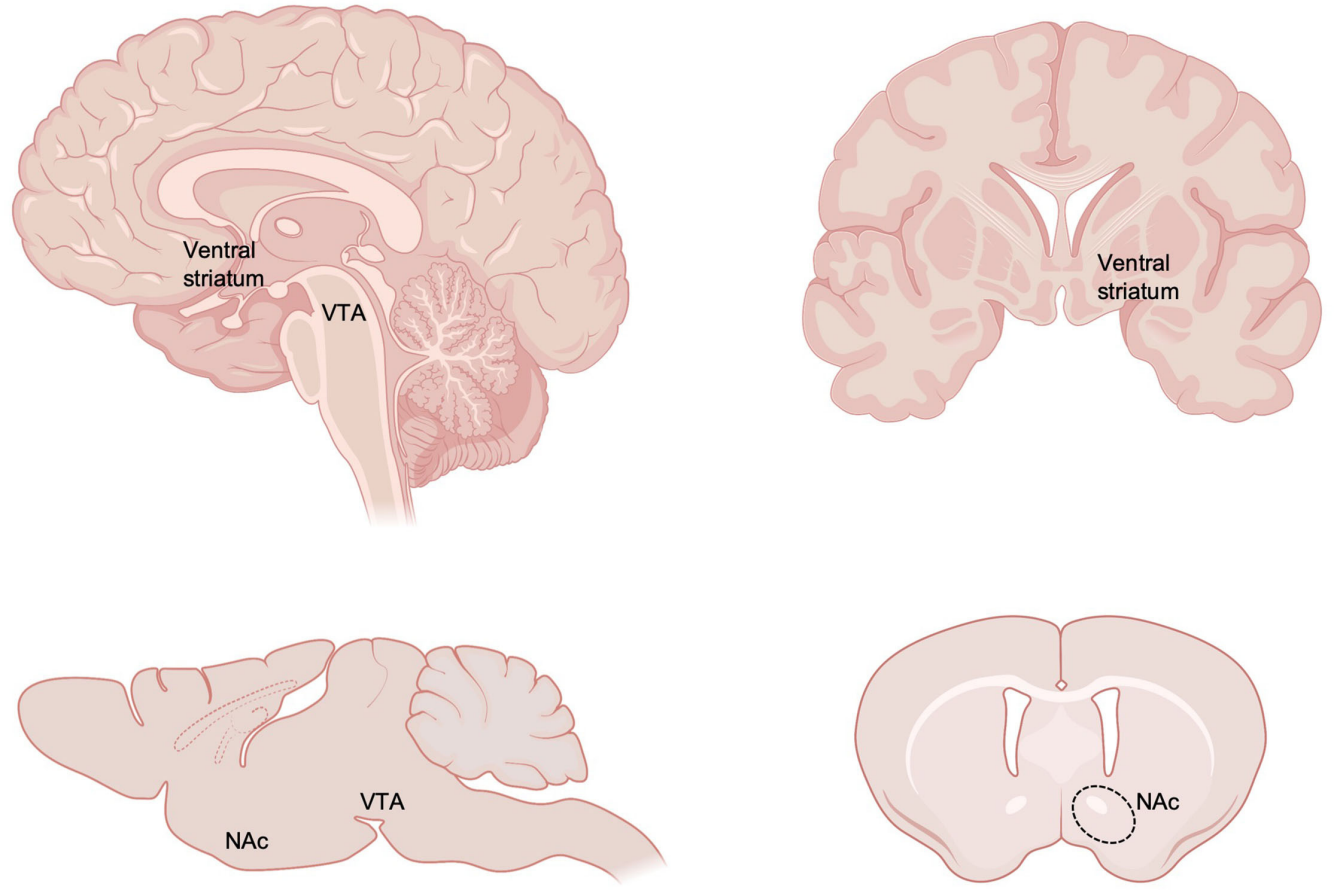
Neuroanatomy of key reward circuitry in humans and rodents. The current review focuses on the impact of early life stress on reward circuitry, with a particular focus on ventral tegmental area (VTA) and nucleus accumbens (NAc), part of the ventral striatum. Created with https://www.BioRender.com.

The robust impact of ELS on increased risk for mood and substance use disorders implicates enduring alterations within reward and motivation circuitry of the brain, which functions at the intersection of negative and positive valence domains implicated in the pathophysiology of these disorders (17–23). The brain's reward circuitry is classically comprised of the ventral tegmental area (VTA) and its dopaminergic projections to the nucleus accumbens (NAc, part of the ventral striatum; Figure 1) and other forebrain targets including prefrontal cortex (PFC), amygdala, and hippocampus. In addition to dopaminergic modulation from VTA, the NAc also receives dense glutamatergic innervation from these same forebrain regions and serves as a central integration point for cognitive and executive input from PFC, emotional information from amygdala, and contextual and emotional input from hippocampus (24–28). Imbalance of these systems is strongly associated with mood, psychiatric, and substance use disorders (19, 29, 30). GABAergic medium spiny neurons of the NAc project locally as well as to VTA, ventral pallidum, bed nucleus of the stria terminalis, lateral septum, amygdala, and lateral hypothalamus among other regions to ultimately regulate motivated behavior. Neurons of the VTA, NAc and broader reward circuitry undergo protracted development and continue to mature through adolescence which may leave them vulnerable to early environmental insults (31–40). Here, we review the enduring impact of ELS on reward circuit function, connectivity, and molecular development in humans and rodents, with a focus on NAc.

## Impact of Early Life Stress on Human Reward Processing and Connectivity

A growing body of behavioral and neurobiological studies in humans suggest ELS-related alterations in reward-processing and connected brain circuitry. Behaviorally, children and adolescents exposed to ELS show challenges in aspects of reward processing and decision-making. There are multiple reports of lower reward responsivity and approach motivation in youth exposed to physical abuse (41, 42), social neglect (43, 44), and other adverse childhood experiences (42, 45–47). In such work, participants often need to make speeded responses to a target to receive rewards or positive feedback. Looking at different aspects of reward processing and decision-making, data suggests slower learning about rewards after adversity. For example, physical abuse and maltreatment was related to slower reward learning over time and an insensitivity to the expected value of a reward (41, 48); this fits with similar results noting lower reward learning after social neglect (44). Such patterns have also been noted in adult samples where adversity was retrospectively reported. Pechtel and Pizzagalli found women who suffered sexual abuse as children had lower behavioral accuracy when using learned information during a reward task (49). Interestingly, impaired reward learning from childhood institutionalization can be rescued through intervention with high-quality foster care between 6 and 33 months of age, implicating some plasticity through early childhood (44).

ELS-associated reward and decision-making differences are driven by a combination of high levels of risk-taking, insensitivity to different valences of feedback, and other processes. Maltreated children take excessive risks during different decision-making tasks (48, 50). In particular, youth exposed to adversity, such as maltreatment, have difficulty avoiding losses (48, 51) and make more impulsive choices (52–54). In addition, adults who reported high levels of early life adversity showed decreased positive feedback sensitivity during learning (55), and stress-exposed adolescents have challenges in initially learning as well as updating reward contingencies (45). These effects may be mechanistically due to lower feedback-related brain responsivity [measured by event-related potentials; (49)].

Functional MRI (fMRI) has been used to examine neural correlates of reward processing. Early adversity exposure has been found to lower activity levels in portions of the mesocorticolimbic circuit including ventral striatum during different reward-related tasks, such as anticipating monetary gains. For example, adolescents exposed to early social neglect had lower ventral striatal and caudate fMRI responses during the anticipation of rewards (56). These findings are consistent with studies that found lower ventral striatal activity in youths exposed to emotional neglect (57) and youth presenting with attachment issues after maltreatment (58). Moreover, blunted development of reward-related ventral striatum activity partially mediated the association between emotional neglect and greater depressive symptomatology (57). Lower ventral striatal activity has also been found when using rewarding *social* stimuli (i.e., happy faces) (59). These patterns persist in adults with a history of early adversity: high levels of adversity in childhood and adolescence blunted reward-related ventral striatum responses in adulthood (60–62). In addition to the ventral striatum, higher childhood adversity has been linked to lower activation in the putamen for adults during the anticipation of potential rewards (63), as well as potential losses (64). Other studies, however, reveal a more complex picture. Lower adolescent neighborhood quality, a contextual variable likely correlated with adversity, was associated with greater average fMRI activation in the ventral striatum during the anticipation of monetary gains (65). Early adversity was also found to improve learning from positive outcomes, but also led to impulsive decision-making, both of which were mediated by ventral striatal responses (52).

In addition to task-related activity, ELS has been found to alter resting-state connectivity of reward circuitry. Child maltreatment and early institutional care was associated with increased coupling between ventral striatum and regions of the PFC (66, 67), and socioeconomic disadvantage was associated with increased coupling between ventral striatum and PFC and cerebellum (68). Including broader reward circuitry, early adversity and socioeconomic disadvantage were also recently found to blunt development of VTA-PFC development (69) and reduce VTA-hippocampal connectivity (70).

These functional consequences of ELS may stem from altered brain structure and development, including mesocorticolimbic reward circuitry. For example, in a large cohort of adults (*N* = *3036*), higher childhood adversity exposure was associated with smaller caudate volumes (71). Childhood trauma is also related to lower white matter integrity in fiber tracts connecting the caudate, ventral striatum, and prefrontal cortex (46, 72, 73). However, rodent models of ELS (discussed below) suggest changes in reward circuitry are at the level of excitatory and neuromodulatory signaling rather than gross structural changes. Focusing on connectivity may provide unique information about the impacts of ELS, explaining different aspects of the effects of ELS compared to the task-based activation studies reviewed above.

Of note, there are multiple open questions related to this research area, suggesting many important future research directions in human samples. First, while many projects conceptualize adversity as impacting mesocorticolimbic and reward-related processes, it is possible, instead, that the interaction between adversity and neurobiology could be diagnostic of resilience after stress exposure. Some studies have found childhood adversity was not related to mesocorticolimbic functional activity *per se*, but rather reduced activity - *in the context of high levels of adversity*- predicted poorer mental and physical health (74–76). For example, high early adversity in combination with lower ventral striatal responses to reward was related to more depression symptoms (74). Second, it is not yet clear how adversity might impact brain areas outside of the mesocorticolimbic circuit that may be related to reward processing. Conflicting results have noted lower (45, 77), as well as higher activity (78), in portions of the prefrontal cortex including the middle frontal gyrus and subgenual cingulate. Finally, it is still necessary to refine and clarify how reward-related processes are impacted by adversity. As noted above, there is ongoing debate about whether adversity may influence the processing of rewards or punishments, as well as brain activity during the anticipation or the consumption of rewards, although animal studies are providing insights into these questions. Relatedly, research groups have also noted lower mesocorticolimbic reactivity specifically for social, as opposed to monetary, rewards (79, 80). Rich experimental paradigms aimed at decomposing these different processes will be critical to move the field forward.

## Impact of Early Life Stress on Motivated Behavior and Reward Circuit Activity From Non-Human Animal Models

### Brief Overview of Rodent Models for Studying ELS

Lack of access to brain tissue, experiential and genetic heterogeneity, and ethical constraints on conducting studies with humans have underscored the need for non-human animal models for understanding the neurobiological consequences of ELS. Researchers have modeled ELS in rodents using a variety of paradigms, including separation of pups from their mothers (typically done in the first two wk of life for 3–4 hours/day, with or without early-weaning); a single bout of prolonged (24-h) maternal deprivation; or limited bedding and nesting resources (LBN) provided in the home cage (81, 82). The types of human ELS that these paradigms model are not entirely clear, however. On the surface, maternal separation or deprivation paradigms appear to model neglect (lack of access to or failure of the caregiver to provide adequate care and address the needs of offspring). However, studies have documented that dams increase nursing and grooming time with pups upon reunion, potentially to compensate for lost time nursing during separation (83), and the cognitive and emotional consequences of rodent maternal separation or deprivation are unknown. Limited bedding and nesting paradigms have been described as modeling a lack of resources, erratic, dysfunctional, or fragmented maternal care, or mimicking aspects of abusive behavior – which span dimensions of deprivation and threat (84–87). An important consideration is how human and rodent developmental timing of reward circuitry aligns. Direct comparison of rodent and human reward circuitry development in the neonatal periods is lacking, although it is suggested that rodent brain development is shifted relative to human development such that the first week of rodent life is approximately aligns with the last trimester of human gestation (16, 88). While a majority of rodent models of ELS begin ELS in the first few days after birth (86, 89), some shift stress to start around P9-10 (82, 90, 91). Each of these models of ELS have been documented to alter pups' plasma corticosterone levels acutely (85, 92, 93), although the long-term impact on basal and stress-induced corticosterone appear to depend on type and timing of stress (reviewed in (86). Each of these paradigms has also been shown to alter offspring defensive, depression-like, and reward-related behaviors, although again type and timing of stress have distinct impact, and sex differences have been documented (82, 89, 94–99).

### Motivated Behaviors

Rodent models of ELS have identified alterations in motivated and reward-seeking behavior, as well as alterations in physiological functions in the reward circuitry (23, 96, 100–102). ELS impacts motivation for natural rewards, although the literature employing natural rewards in behavioral tasks is scant compared to studies with drugs of abuse (reviewed below), and currently available studies paint a complicated picture, particularly with respect to sex differences in behavior. In one study, maternal separation ELS decreased lever bar pressing for a sucrose solution in male Wistar rats (103), indicating that ELS reduces motivation for palatable rewards. Other studies assessing sucrose preference in a free-choice model confirm that various animal models of ELS reduce sucrose preference in male rats and mice, interpreted as increased anhedonia (82, 94, 96). However, other studies have found either a sex-specific effect of ELS on sucrose preference in female rats (104), no effect in female mice (95), or no effect at all (105, 106). When effects of ELS are found, the general result is that it reduces motivation for food rewards.

ELS also alters other natural reward-directed behaviors in rodent studies. Overall, ELS (maternal separation or LBN) appears to reduce conditioned place preference for a palatable food reward (107), although there may be a latent impact of ELS where this effect only emerges across development and is stronger in females (108, 109). However, another study reported opposite findings in which female Sprague-Dawley rats exposed to LBN show increased motivation for food reward in a one-h free access task (106). To our knowledge, only one study has assessed the effect of ELS on motivation for access to a social reward (110). In this study, LBN increased sexual motivation in both male and female Long-Evans rats (110). Whether ELS also increases other forms of social motivation is not yet known. Taken together, these findings suggest that the effects of ELS on motivation for natural rewards are dependent on many factors including the type of reward, sex, age, species/strain, and the type of adversity experienced during early life (i.e., forced maternal separation vs. disrupted maternal care due to limited resources). More studies are needed in order to tease apart how these various factors are differentially contributing to the effects of ELS on motivational drive for food and social rewards.

The majority of work assessing the effects of ELS on motivated behavior have focused on motivation for drug rewards such as psychostimulants, ethanol, and opiates. A consistent finding is that ELS increases self-administration of various psychostimulants. For example, maternal separation increases active lever responses as well as the number of infusions of methamphetamine in male Long-Evans rats (111, 112). Studies focused on cocaine largely find that ELS increases cocaine-seeking behaviors. Maternal separation combined with early weaning at P17 increases reward-seeking behavior and self-administration for cocaine in male and female CD1 mice (113). LBN results in higher acquisition of cocaine self-administration in Sprague-Dawley rats without increasing daily cocaine administration (107), suggesting that ELS is altering novelty-seeking behavior for cocaine. ELS from P14-21 in CD1 mice alters cocaine conditioned place preference and relapse behaviors (100). Additionally, LBN rats show a reduced hedonic set point for cocaine, indicating that while they are motivated to lever press for cocaine administration, they do not find it as rewarding as control rats. These differences observed in behavioral responses are likely related to these assays examining different aspects of reward-seeking behavior (i.e., assessing “liking” behavior versus assessing “wanting” behavior) (114, 115).

Many studies report that ELS increases reward-seeking for ethanol. Maternal separation rearing increases both lever pressing and consumption of ethanol in male Swiss ICR mice (116) and male and female Sprague-Dawley rats (117, 118). Additionally, maternal separation increases ethanol consumption in free-choice tasks in both male C57BL/6 mice (105) and male Wistar rats (119). Similarly, increases in ethanol consumption have been reported in maternal deprivation reared male and female Sprague-Dawley rats (120). These reports indicate that ELS models increase both motivated “wanting” and hedonic “liking” behaviors for ethanol. However, inconsistent findings have also been reported. Maternal deprivation reared male and female Wistar rats do not show increased ethanol intake until after two-wk of withdrawal and additional stress in adulthood (121), and LBN rearing decreases ethanol consumption during acquisition in male but not female C57BL/6J mice (122). In this same study, ethanol consumption levels eventually matched between groups, suggesting that ELS reduced the rewarding properties of ethanol only during early exposure and did not cause a long-lasting deficit in preference.

Conflicting results have been reported regarding the impact of ELS on motivation for opioids. In male Wistar rats, maternal separation induced place preference at a lower dose of morphine than in controls (123). A study with female Sprague-Dawley rats reported that LBN did not affect opioid self-administration itself, but rather it increased relapse-like behavior for heroin and remifentanil, and increased motivation for remifentanil (106). These data suggest an ability for LBN to increase vulnerability for opioid misuse following LBN in females. Males were not tested, so it is unclear whether they would also display this vulnerability. Another study including both male and female Long-Evans rats, similarly, found no effect of LBN on acquisition of morphine self-administration in females (124). However, LBN males administered less of a low dose of morphine than control reared males, and administered less morphine on a progressive ratio schedule, suggesting they were less motivated to take morphine at this dose. Consistent with a protective effect of LBN against this addiction-related phenotype in males, LBN reduced impulsive choice, a behavior associated with substance use disorders and mediated, in part, by the NAc (124–126). In contrast, no effects of LBN on motivation for morphine or impulsive choice were observed in females (124). The inconsistent effect of LBN on motivation for opioids in females may be due to the use of different drugs, a focus on different outcome measures, or due to differences in the implementation of the LBN model.

### Reward Circuit Activity and Function

Collectively, these behavioral studies reveal that ELS can affect motivated behavior that is mediated by reward circuitry, albeit to varying degrees based on a variety of factors, and suggest that ELS alters reward circuit function. Dopamine release from VTA modulates NAc signal integration and activity and is necessary for the rewarding properties of both natural rewards and drugs of abuse (127, 128). Dopamine signaling has been associated with incentive-salience of rewarding and addicting stimuli (129) and reward prediction-error (130, 131), as well as signaling aversive and stressful stimuli (21, 132, 133). ELS has been found to alter VTA morphology, decrease GABAergic inhibition onto dopamine neurons, increase excitability of dopaminergic neurons in VTA, increase baseline dopamine levels released from VTA into NAc, and alter dopaminergic response to stressors (90, 134–142). However, a meta-analysis evaluated the effects of ELS on biochemical indicators of dopamine signaling and found that while ELS causes a consistent and robust increase in the dopamine metabolites DOPAC and HVA, there may only be a small increase in dopamine itself in the striatum (143). Consistent with these findings, maternal separation increases dopamine turnover (ratio DOPAC/DA) in the NAc of adolescent CD1 mice, an effect increased by exposure to cocaine (144). In addition to dopamine metabolism, dopamine clearance regulates the amount of dopamine in the synapse. Maternal separation decreased the rate of dopamine clearance in the spontaneously hypertensive rat (SHR), a model of attention deficit hyperactivity disorder (145). These studies could suggest ELS impairs dopamine transporter (DAT) function, or this result may be specific to the SHR model. Other studies suggest maternal separation ELS increases cocaine-induced but not baseline DAT levels in the NAc of adolescent CD1 mice, although other aspects of DAT function were not tested (144). Together, these studies indicate that ELS alters tonic and/or stimulus-induced dopamine from VTA to NAc, and that enduring alterations in NAc processing may in part be due to altered dopamine clearance.

Consistent with this idea, there is evidence that ELS alters NAc physiology. LBN decreases presynaptic glutamate transmission, as indexed by a reduction in the frequency of spontaneous excitatory postsynaptic currents (sEPSCs), although only in male rats (124). This effect is consistent with a study that found maternal separation decreased GluA2 AMPA subunit expression in the NAc of males but not female rats (146). The ratio between AMPA and NMDA receptors is a key factor governing glutamatergic plasticity. Morphine increases the AMPA/NMDA ratio in males (147) and LBN prevents this effect (124). Thus, in conditions where ELS reduces opioid self-administration, resilience is linked to a blockade of opioid-induced glutamatergic plasticity in the NAc.

An important question in neuroscience is the cellular specificity of effects. MSNs are the primary neuronal cell type of the striatum. MSNs are GABAergic and subclassified by whether they express D1- (*Drd1*-expressing) or D2- (*Drd2*-expressing) type dopamine receptors, which use distinct intracellular signaling cascades and have opposing effects on activity. D1-type receptors are coupled to Ga_s_ and Ga_olf_ G-proteins and binding of dopamine leads to increased adenylyl cyclase activity, increased cyclic adenosine monophosphate (CAMP) production, and activation of protein kinase A (PKA) (148, 149). D2-type receptors have 100-fold higher affinity for dopamine and are instead coupled to Ga_i_ and Ga_o_ proteins, and dopamine binding leads to decreased CAMP and PKA activity. D1-MSNs are thought to be preferentially activated by phasic dopamine bursts, while D2-MSNs — which have greater baseline cellular activity—are thought to have greater sensitivity to tonic dopamine release (150). It is not yet known how ELS alters activity of D1 and D2 MSNs, although differential involvement of these cell types may well mirror their response to adult stress. Chronic stress in adult male mice decreases excitatory transmission onto D1-MSNs, causes D1-MSN long-term depression, and increases the threshold to excite D1-MSN activity (151, 152). Activating D1-MSNs after chronic adult stress alleviates depression-like behavioral changes in male mice. In contrast, chronic adult stress increases excitatory transmission onto D2-MSNs without altering their excitability, and activation of D2-MSNs increases susceptibility to later chronic adult stress (151).

Overall, these findings suggest that reward circuit physiology is particularly vulnerable to the effects of ELS, likely due to the ongoing maturation of dopaminergic reward circuitry in the postnatal period when these ELS manipulations are being implemented (31–40).

## Impact of Early Life Stress on Transcription and Epigenetic Regulation Within NAC

### Genome-Wide Transcriptional Alterations

The impact of early life stress on functional activity in NAc likely arises from changes in the molecular development of VTA, NAc, and broader reward circuitry. Genome-wide expression changes in reward circuitry resulting from ELS have been surveyed by RNA-sequencing. RNA-seq provides an opportunity not only to look for gene expression changes in an unbiased manner, but also to examine broad patterns of change that would be impossible to observe when sampling only a few candidate genes. ELS consistently induces changes in reward circuitry gene expression that last into adulthood in male and female mice (94, 95, 100, 124, 153). Interestingly, two studies comparing male and female transcriptomic responses to ELS (using different types of stress and at different juvenile states) have found around twice as many genes altered in female than male NAc (95, 124). Indeed, both baseline and stress-induced sex differences in the transcriptome have been described in NAc (95, 154–156). One interesting question is how prior ELS alters gene expression response to future salient experience such as additional stress or drugs of abuse. The transcriptomic response to adult stress is highly dependent upon whether or not a mouse experienced prior ELS: expression of more genes in both male and female NAc were altered after adult stress if mice had previously experienced early life stress. Moreover, the transcriptional response to adult stress showed opposite regulation in ELS vs. standard reared female mice in VTA and NAc (95). Genome-wide analysis also revealed that a subset of genes in male and female VTA and NAc did not have altered expression in response to ELS alone, but instead were *primed* by ELS such that latent expression changes were only revealed by later adult stress (95). The impact of ELS on genome-wide response to adult stress may be dependent on sex and the timing of stress. The pattern of increased transcriptional changes in response to adult stress given prior ELS from P10-17 was also observed in female (but not male) VTA and PFC which both send inputs to NAc (95). In male PFC, ELS alone predominately down-regulated gene expression, which may blunt response to future stimuli (153). Similarly in hippocampus, early postnatal stress (from P2-12) and adolescent stress (from P38-49) blunted transcriptomic response to acute adult stress in mice and rats (157, 158). Transcriptomic analyses also revealed that one potential mechanism for ELS-induced sensitivity to stress and drugs of abuse in male mice may be through altered plasticity. In VTA, ELS reduced transcriptional programs downstream of the transcription factor OTX2 (94), which has been identified as a key regulator of critical period plasticity (159–161). Transiently reducing *Otx2* in VTA in a late postnatal sensitive period for stress exposure mimicked the effects of ELS on sensitivity to future stress experience and depression-like behavior, while restoring *Otx2* levels in this sensitive period rescued the effects of ELS (94). In contrast, ELS may inappropriately preserve plasticity in NAc, including changes in genes associated with critical period plasticity and synaptic development (94, 95, 100). In NAc, a second stress exposure reversed such plasticity signatures which may in turn decrease physiological plasticity and lead to behavioral inflexibility and maladaptive coping behavior (95). Reduced plasticity in response to later salient exposures extends beyond stress to response to drugs of abuse as well: cFos (a marker of neuronal and gene expression activity) in NAc was reduced after ELS and cocaine exposure plus reinstatement (100).

### Alterations in Target Genes

Examining the effect of ELS on specific candidate genes and proteins has identified a number of key mechanisms through which ELS alters motivated behaviors and stress response. Release of BDNF from VTA and binding to TrkB receptors in NAc is necessary for a depression-like response to adult stress (133) and physiological and behavioral response to reward and drugs of abuse (162–164). Blocking BDNF/TrkB signaling in NAc during exposure to ELS in mice (maternal separation from P3-14) simultaneously restored sucrose preference and sensitized behavioral response to chronic unpredictable adult stress on the open field test (165), indicating that the enduring and opposite consequences of ELS on motivated and anxiety-related behavior are regulated by transient developmental alterations in BDNF-TrkB signaling. ELS also increased expression of cannabinoid 1 receptors (*Cb1r*) and FK506-binding protein (*Fkbp5*) in NAc, which was further linked to alcohol consumption in rodents (119, 166). ELS in the form of predator odor exposure from P1-3 decreased expression of mu and kappa opioid receptors (*Orpm1* and *Oprk1*, respectively) in female but not male NAc acutely on P3, although *Orpm1* expression rebounded and was significantly higher than control females by postnatal day 33 (167). Mu opioid receptors contribute to euphoric and analgesic properties of opioid use and development of drug tolerance, which could explain a lack of motivation to lever-press for opioids in self-administration tasks (106, 124). Finally, ELS (LBN in male mice) downregulated the alpha-2 subunit of GABA_A_ receptors and reduced frequency of miniature inhibitory postsynaptic currents in NAc, which was associated with both enhanced behavioral response to acute cocaine and blunted sensitization to repeated cocaine exposure (168).

ELS can also alter expression of dopamine D1-type (*Drd1*) and D2-type (*Drd2*) receptors themselves, which alters striatal response to stimuli. However, the direction of change is not consistent across reports. In one study, maternal separation ELS reduced NAc levels of *DAT, Drd1*, Drd2, and *Drd3*, which were correlated with reduced spatial learning in these rats (169). Other studies have confirmed reduced *Drd1* (108) and *Drd2* (170) receptor expression in the NAc of female mice which can reduce dopaminergic drive. In contrast, other studies in males found *Drd1* expression in the NAc to be unaffected by ELS (108, 170). Maternal separation increased NAc *Drd2* in males (119, 144, 171), an effect which was accompanied by a stress-induced increase in dendritic morphology in the NAc (171). Together these studies suggest sex-specific impacts of ELS on dopamine receptors in NAc, although additional research is needed to confirm sex-specific effects directly and determine the contributions of type and timing of ELS.

### Epigenetic Alterations

Gene expression is regulated by a complex interaction between transcription factors and epigenetic regulatory mechanisms which fine-tune when and to what extent genes are expressed without altering the genetic sequence itself (172). DNA methylation – the addition of a small methyl group typically to the 5^th^ carbon of cytosine residues (5mC) – is associated with suppression of gene expression in gene promoters, with mixed effects on transcription in other genomic regions (173). *De novo* DNA methylation is accomplished by the enzyme DNA methyltransferase 3A (DNMT3A) and DNMT3B, while maintenance methylation (across cell division) is accomplished by DNMT1. Maternal separation for three h daily from P2-15 increased overall *Dnmt1, Dnmt3a*, and *Dnmt3b* expression in the NAc, increased methylation at several target genes of interest (protein phosphatase catalytic subunit 1c and adenosine receptor 2A, vesicular glutamate transporter 3), and decreased their expression (174, 175). These target genes are involved in neuronal plasticity and response to cocaine, and may be a mechanism for ELA-induced hypersensitized response to cocaine (174). ELS also increased baseline levels of Methyl CpG binding protein 2 (MeCP2) in NAc (112, 175), a protein that binds or “reads” methylated DNA and is involved in gene silencing, although the specific genes differentially bound by MeCP2 after ELS are still unknown. Interestingly, MeCP2 is also elevated in NAc after self-administration of methamphetamine and ELS significantly blunts this effect, which is functionally related to increased methamphetamine self-administration among ELS-exposed rats (111, 112). Alterations in DNA methylation have also been identified as potential mechanisms of ELS-induced changes in gene expression of dopamine receptors and other target genes in NAc, including hypermethylation of the *Drd1a* promoter region in females (108).

Gene expression is also influenced by interactions between DNA and the histone proteins around which it is wrapped (176). Post-translational modifications to histone protein tails – including acetylation, methylation, and other modifications – can increase or decrease compaction, and repress or allow gene expression, respectively. 24-h maternal deprivation at P9 ELS has been shown to alter histone acetylation in VTA, which is directly linked to altered GABAergic function in VTA and increased dopaminergic excitability, and which is restored by histone deacetylase inhibitor treatment (90, 91, 141, 177). ELS (combined maternal separation and limited nesting material from P10-17) also broadly altered levels of post-translational histone modifications in NAc across postnatal development in a sex-specific manner (178). Most notably, ELS reduced mono- and di- methylation of histone 3 lysine 79 (H3K79) in adult male and female mice. The enzyme that “writes” H3K49 methylation, *Dot1l*, was also upregulated by ELS in adult male and female NAc, specifically within D2-MSNs (178), consistent with a role for D2-MSN activation increasing susceptibility to subsequent stress (151). H3K79 methylation is generally associated with enhanced transcription and genomic stability, and indeed both ELS and *Dot1l* overexpression predominately downregulated gene expression in NAc. Moreover, D2-specific overexpression in NAc recapitulated the impact of ELS, while knockdown or small-molecule inhibition of *Dot1l* ameliorated the impact of ELS on susceptibility to adult stress (178). Finally, ELS alters histone turnover dynamics in NAc. ELS results in faster accumulation of the replication-independent histone H3.3 variant in NAc across development, which is associated with increased susceptibility to adult stress, and reversing this phenomena can rescue depression-like behavior in mice (179). These epigenetic changes in reward circuitry following ELS underlie enduring changes in gene expression, and can also help explain latent changes in gene expression in response to future stressful or rewarding stimuli. These molecular mechanisms ultimately impact physiological functioning of reward circuitry, altered response to stressors and rewards, and risk for psychiatric disease.

## Discussion

Looking holistically, there is growing evidence that ELS influences behavior (Table 1) and neurobiology (Table 2) involved with reward processing, with a focus here on NAc. However, additional work is critically needed at multiple “levels of analysis” (e.g., human systems neuroscience; non-human physiology, etc.) to increase understanding regarding the connections between ELS and later negative outcomes. Below, we elaborate on future directions for those working with human and preclinical samples, focused on different constructs connected to the review.

**Table 1:** Significance of evidence for the effects of ELS on motivated behaviors in human and rodent model studies.

**Behavior**		
Rodent	Increased Motivation for Alcohol	STRONG EVIDENCE[7 Supporting Studies: Refs. (105, 116–121)/1 Against: Ref. (122)]
	Increased Motivation for Psychostimulants	STRONG EVIDENCE[5 Supporting Studies: Refs. (100, 107, 111–113)]
	Increased Motivation for Social Rewards	MODERATE EVIDENCE[1 Supporting Study: Ref. (110)^**^]
	Reduced Motivation for Food Rewards	INCONCLUSIVE EVIDENCE[7 Supporting Studies: Refs. (82, 94, 96, 103, 107–109)/4 Against: Refs. (95, 104–106)]^*^
	Increased Motivation for Opioids	INCONCLUSIVE EVIDENCE[2 supporting studies: Refs. (106, 123)/1 Against: Ref. (124)]
Human	Lower Approach Motivation (Self-Report & Behavioral)	STRONG EVIDENCE[6 Supporting Studies: Refs. (42, 47, 49, 50, 63, 78)]
	Lower Reward Learning Over Time	STRONG EVIDENCE[7 Supporting Studies: Refs. (41, 43–45, 48, 52, 55)]
	Impulsivity and Excessive Risk-Taking	MODERATE EVIDENCE[3 Supporting Studies: Refs. (48, 51, 53)]

* *Sex differences present in results of studies;*

** *To our knowledge, the only study to assess the impact of ELS on a social reward*.

**Table 2:** Significance of evidence for the effects of ELS on reward circuit neurobiology in human and rodent model studies.

**Neurobiology**		
Rodent	Increased Variability in NAc-VTA Connectivity and Dopamine Clearance	STRONG EVIDENCE[12 Supporting Studies: Refs. (90, 134–142, 144, 145)/1 Against: Ref. (143)^*^]
	Long-lasting Changes in Transcriptome Expression	STRONG EVIDENCE[6 Supporting Studies: Refs. (94, 95, 100, 124, 153, 156)]
	Alterations in DNA Methylation	STRONG EVIDENCE[5 Supporting Studies: Refs. (108, 111, 112, 174, 175)]
	Decreased Glutamatergic Transmission and Receptor Expression	MODERATE EVIDENCE[2 Supporting Studies: Refs. (124, 146)]
	Altered D1 and D2 Receptor Expression	MODERATE EVIDENCE[6 Supporting Studies: Refs. (108, 119, 144, 169–171)/2 Against: Refs. (108, 170)]
	Altered Histone Acetylation and Histone Turnover Dynamics	MODERATE EVIDENCE[2 Supporting Studies: Refs. (178, 179)]
Human	Lower Functional Activity in Ventral Striatum (or other portions Basal Ganglia)	STRONG EVIDENCE^**^[10 Supporting Studies: Refs. (56–62, 64, 65, 79, 80)/1 Greater Activity: Ref. (65)]
	Heightened Resting Functional Connectivity (from Striatum to mPFC)	MODERATE EVIDENCE[2 Supporting Studies: Refs. (66, 68)]
	Reduced White Matter Connectivity (from Striatum to mPFC)	MODERATE EVIDENCE[3 Supporting Studies: Refs. (46, 72, 73)]
	Smaller Volumes in Ventral Striatum (or other portions Basal Ganglia)	MODERATE EVIDENCE[1 Supporting Study, but *large N:* Ref. (71)]
	Smaller Volumes in Broader Corticostriatal Circuit (OFC)	MODERATE EVIDENCE[3 Supporting Studies, Refs. (180–182)]

* *Results of a meta-analysis*

** *Unclear if brain activity is lower to positive feedback or greater to punishment, or insensitivity to change in valence*.

### Operationalization and Categorizing Life Stress Across Early Development

In considering human and non-human research focused on ELS, it will be critical to think about rich characterizations of stressful life experiences. Here, we took a more broad and inclusive definition of ELS, but there is a great deal of active inquiry focused on the phenomenology of ELS. For example, in humans, there is an interest in considering the interactions, “lived experiences”, and subjective perceptions of different forms of ELS. Such factors may be important to consider, but difficult to capture in both human and non-human research. Clear from numerous epidemiological studies is that 60% of individuals reporting one form of ELS will experience two or more forms of adversity before reaching adulthood (5–7, 183). Interestingly, regardless of whether objective (court) records suggested maltreatment or ELS, subjective reports of ELS more robustly predicted psychopathology (184). Limited work, to our knowledge, has attempted to richly probe these aspects of ELS, but all of these elements will likely influence neurodevelopment and may impact trajectories of mesocorticolimbic neurobiology.

There are multiple approaches to modeling ELS and the field must strike a balance between more mechanistic approaches and the common co-occurrence of different forms of ELS. Researchers in epidemiology have been drawn to cumulative exposure models that sum up the total number of adversities suffered. This framework has nice connections to allostatic load and other neurobiological frameworks, has high explanatory power, and can deal with the common pattern of co-occurrence between many forms of adversity (185–187). However, these models provide less clarity about potential mediating mechanisms. More recently, starting frameworks (10, 188) argue for the examination of differences between dimensions of adversity (i.e., harshness vs. unpredictability; deprivation vs. threat) to advance mechanistic understanding of the impact of ELS. Further complicating our understanding, while the negative impacts of early life adversity are well-known and well-documented, many people exposed to early life adversity do not develop illnesses, and are, instead, protected from later stressful experiences (189, 190). A burgeoning body of research suggests that adversity, in modest amounts, can lead to the subsequent development of resilience (191–193). For example, non-human primates exposed to challenging, but not overwhelming, stressful events early in life show less anxiety and lower hypothalamic–pituitary–adrenal axis responsivity later in life (194). Similarly, moderate but not extreme levels of early adversity are related to larger volumes and greater activity in the prefrontal cortices of non-human primates (195, 196). Some implementations of LBN in rats leads to resilience after facing adolescent stress (197), adult stress (193) and reduced morphine self-administration (124), consistent with a “stress inoculation” model. However, it is clear that even with controlled rodent models of ELS such as LBN there is wide range in whether studies find evidence of inoculation or sensitivity to additional stress, drugs, or other stimuli. Identifying factors that contribute to vulnerability vs. sensitivity is crucial and will aid in targeted implementation of treatments for stress-related diseases.

Relatedly, one major translational challenge in most preclinical studies is that nearly all of our ELS models do not adequately capture the full “transactional nature” of stress [e.g., (198)]. Put another way– ELS is related to cognitive, affective, physiological, and neurobiological changes, and these alterations may affect how individuals respond to subsequent life stressors (199). In humans, there is rich support for so-called “stress sensitization” or “two-hit models”. For example, women with exposure to one or more childhood adversities (e.g., family violence, parental psychopathology) were more likely to become depressed by a lower “dose” of total stress than women without such adversity (200). This is true for depression, anxiety, and PTSD, and found during childhood, adolescence, and adulthood (7, 200–206). Connected to this, Hanson, Knodt, Brigidi & Hariri (2018) found reward-related functional connectivity between the ventral striatum and the medial prefrontal cortex was heightened after the occurrence of both early and then more contemporaneous life stress (67). These functional changes then linked stress exposure to depressive symptoms. Non-human animal work has likewise found evidence of stress-sensitization behaviorally, within reward circuitry, and in other brain systems (82, 94, 95, 207–210). Moving forward, there is a true need to construct complementary experiments in human and non-human samples, focused on similar neurobiological circuits, with interactive discussion between teams leading work in each species. This could aid in understanding these transactional elements, as well as modeling of ELS.

A number of connected open questions remain on “*how*” ELS shapes neurobiology, with interest in direct vs. indirect impacts. Direct impacts may be thought of as emerging due specifically to the elements of ELS, vs. indirect influences being related more to cascading influences of ELS. For example, while unlikely to be purely Hebbian in nature, mesocorticolimbic changes in physiology, transcription, or epigenetic expression could be due to the absence or inconsistency of (“direct”) environmental inputs. In contrast, environmental inputs could activate the HPA axis, causing the release of cortisol and related neuroendocrine messengers. Cortisol is known to be neurotoxic in high and chronic conditions (reviewed in reference (211) and this could (“indirectly”) lead to changes in multiple aspects of mesocorticolimbic neurobiology. Similarly, ELS can alter gonadal hormone levels, such as estradiol, which are potent modulators of reward circuitry (212). In line with recommendations from Callaghan et al. (2019), it is and will be critical to consider the developmental ecology and “goals” of an organism (e.g., attachment; independence) and how ELS may impinge upon these elements to create risk for negative life outcomes (213).

### Considering Underexplored Moderators: Sex and Developmental Timing

As we continue to focus on the impact of ELS, it will be important to think about underexplored moderators and factors that may be driving inconsistencies in findings to date. Two clear factors are the developmental timing of specific stressors and potential sex differences in stress effects.

Developmentally, expression and activity of neurotransmitter systems centrally involved with the mesocorticolimbic circuit (i.e., dopamine) exhibit major changes during early life. The exact developmental sequencing is still of debate (214), but a number of reports suggest dopamine receptor expression peaks during the peripubertal period (215–218). As such, there may be differential behavioral and neurobiological consequences depending on the timing of ELS exposure. A powerful example of this comes from non-human research on amygdala neurobiology. Multiple studies have focused on this brain region in stress-exposed, young adult rodents (219); however, many paradigms were not sensitive to the differential developmental impacts of stress (cf. work by Regina Sullivan et al.; (220, 221). Interestingly, Rosenkranz et al. completed many commonly-used stress manipulations (e.g., repeated restraint stress) in animals of different ages and found many critical differences (e.g., number of spontaneously firing neurons vs. firing rates) depending on when in development the stress occurred (222–224).

Related to sex differences, there is now a growing emphasis on sex as a biological variable. Impacts of ELS for males and females may not be uniform and there may be evolutionary and developmental mechanisms of altered development in response to adversity (97). Stress during the prenatal and early postnatal period increases risk in males for neurodevelopmental disorders, such as autism spectrum disorder and ADHD (225). In females, the effect of ELS can be precipitated later in life by changes in fluctuating hormones, as occurring during puberty, pregnancy, and perimenopause, and is more likely to manifest as anxiety and depression (225). In addition, different forms of ELS are not equally distributed for males vs. females. For example, females are approximately 3–4 times more likely than males to suffer forms of sexual abuse (226, 227). Sadly, especially in human samples, few investigations have examined if ELS exerts differential impacts on mesocorticolimbic neurobiology for males vs. females. This pattern is surprising given that Andersen and colleagues have reported significant developmental sex differences in the striatum; specifically, males, but not females, over-produce D1 and D2 receptors in the striatum during development. These excessive receptors are then pruned prior to adulthood, only in males (228, 229). Thoughtful examination of both sex and developmental timing, across preclinical and human studies, could significantly advance our understanding of the neurobiological sequelae of ELA and the mediating connections between ELA and later negative outcomes.

### Expanding Behavioral and Neurobiology Foci to Further Advance Understanding of ELS

As research continues in this space, increased behavioral and neurobiological precision will aid in understanding the sequelae of ELS, and links with poor mental health. Behaviorally, it will be critical to think about richly decomposing and parsing apart different motivational components connected to the mesocorticolimbic circuit. Basic research indicates that the mesocorticolimbic circuit encodes multiple aspects of reward-learning and decision-making processes such as prediction error, estimation of value, and amount and effort discounting (for review, see (230). However, few studies have examined these types of processes in samples exposed to ELS (77). A richer understanding of how the mesocorticolimbic circuit, and specific connected behavioral processes, may be influenced by ELS – in both human and non-human studies - will be crucial moving forward.

Neurobiologically, the preponderance of work reviewed here is centered on the NAc / ventral striatum (noting that these regions are not defined identically) and VTA, but, this brain region is nested in a larger circuit of motor, cognitive, and limbic brain regions, including portions of the medial prefrontal cortex (mPFC), sub-regions of the anterior cingulate cortex, the thalamus, brain stem, and motor cortex (231–233). As we continue to think about links between stress exposure, neurobiology, and mental health, it will be critical to think about this larger network and how variations in multiple portions of the mesocorticolimbic circuit may give rise to complex behavioral alterations (some of which is reviewed elsewhere (22, 23). For example, several portions of the mPFC support reward responsivity, as well as the processing of self-referential and social information (231, 234). In addition, smaller volumes (180–182) and greater activity in portions of the mPFC were seen for individuals exposed to stress, including child maltreatment or extreme family poverty (235, 236). Interestingly, non-human animal data also supports this idea, as helplessness and stress-vulnerability in rodents was associated with enhanced activity in the mPFC (237, 238). In addition to the mPFC, a few groups have been investigating the VTA and how ELS might impact connectivity of this key node in the mesocorticolimbic circuit in humans. Examined collectively, it is likely that there may be unique neural profiles underlying distinctive deleterious effects of adversity. Individuals exposed to early adversity may show differences in task-based activity in the NAc/ventral straitum, mPFC, or VTA, resting-state connectivity between these areas, or other combinations of these neural phenotypes. A great deal of work is needed to interrogate how individuals with each of these neural patterns may differ in forms of psychopathology, as well as psychosocial risk factors connected to psychopathology (e.g., detrimental emotion processing and regulation strategies). Future research should consider the complex relationships between stress exposure and reward-related brain function in establishing novel strategies to predict, prevent, and treat stress-related psychopathology.

### Concluding Remarks

Here, we review multiple connected literatures on the impacts of ELS on mesocorticolimbic neurobiology — spanning human and non-human, and molecular to brain circuits. These bodies of research suggest that ELS may impact critical behavioral and neurobiological processes central to motivation and reward processing. Multiple projects focused on adversity exposure in human and non-human samples suggest decreased responding or inability to use feedback and reward-related cues. Changes in these motivation-related behaviors appear to be mediated by a host of neurobiological changes, including lower functional activity in the ventral striatum, altered glutamatergic signaling, changes in dopaminergic modulation and clearance, and altered molecular signatures of altered brain plasticity that may be regulated by epigenetic mechanisms. While exciting progress is occurring in this space, there are often more open questions about the development and function of mesocorticolimbic neurobiology in relation to ELS. A richer ability to dissect heterogeneity, while integrating stress- and developmental- neurobiology, will be critical to reduce the impacts of ELS and related long-term mental health challenges.

## Author Contributions

All authors listed have made a substantial, direct and intellectual contribution to the work, and approved it for publication.

## Funding

This work was supported by R00 MH115096 (CP), BBRF Young Investigator Award 28627 (CP), NSF Award IOS-1929829 (DB), R01 DA049837 (DB), and T32DA007237 (AW).

## Conflict of Interest

The authors declare that the research was conducted in the absence of any commercial or financial relationships that could be construed as a potential conflict of interest.

## Publisher's Note

All claims expressed in this article are solely those of the authors and do not necessarily represent those of their affiliated organizations, or those of the publisher, the editors and the reviewers. Any product that may be evaluated in this article, or claim that may be made by its manufacturer, is not guaranteed or endorsed by the publisher.
